# Impact of Dietary Supplemental Chromium and Zinc on the Performance of Laying Hens, Egg Quality, and Blood Chemistry

**DOI:** 10.1155/sci5/2691313

**Published:** 2026-07-21

**Authors:** Shadi Sedgh-Gooya, Ahmad Mohebbifar, Mehran Torki

**Affiliations:** ^1^ Animal Science Department, College of Agriculture and Natural Resources, Razi University, Kermanshah 67156-85423, Iran, razi.ac.ir

**Keywords:** blood metabolites, egg production, eggshell thickness, trace minerals

## Abstract

This study evaluated the individual and interactive effects of dietary chromium (Cr) and zinc (Zn) supplementation on productive performance, egg quality, and blood biochemical parameters in Lohmann LSL‐Lite laying hens. A total of 216 hens (42 weeks old) were allocated to a 3 × 2 factorial arrangement with three dietary Cr levels (0, 3, and 6 mg/kg) and two Zn levels (0 and 40 mg/kg) over a 12‐week period. Chromium supplementation significantly affected feed conversion ratio (FCR), hen‐day egg production (EP), and egg mass (EM), with hens receiving no supplemental Cr exhibiting higher EP and EM than those supplemented with 3 or 6 mg/kg Cr (*p* = 0.001). Feed intake (FI) was not influenced by either Cr or Zn supplementation (*p* > 0.05). Both Cr and Zn significantly affected shell thickness (*p* = 0.0259 and *p* = 0.0026, respectively), with Zn increasing and Cr decreasing shell thickness. Chromium supplementation also reduced shell weight (*p* = 0.0138). A significant Cr × Zn interaction was observed for yolk color (*p* = 0.0429), with the highest values recorded in hens fed 40 mg/kg Zn combined with 3 mg/kg Cr. Blood albumin concentrations increased with increasing dietary Cr and Zn levels (*p* = 0.0001), whereas blood triglyceride concentrations decreased following Zn supplementation (*p* = 0.0490). Zinc supplementation increased blood Zn concentrations (*p* = 0.0267), while Cr supplementation reduced blood Cr concentrations (*p* = 0.0001). In conclusion, dietary Cr supplementation at the tested levels did not improve laying performance and, in some cases, reduced EP and EM, whereas Zn supplementation enhanced certain egg quality traits and improved blood biochemical profiles. Furthermore, the interaction between Cr and Zn influenced yolk color and blood Cr concentrations.

## 1. Introduction

Modern high‐producing laying hens require precisely balanced nutrition to maintain health, productivity, and egg quality throughout extended laying cycles [1]. Trace minerals, although required in minute quantities, are essential for metabolic regulation, immune function, and eggshell formation [2]. However, both deficiency and excess may disrupt physiological homeostasis, and existing recommendations [3] may not fully reflect the needs of modern genetically improved strains [4–6].

Chromium (Cr) and zinc (Zn) are trace minerals with distinct yet potentially complementary roles in poultry metabolism [7]. Chromium is associated with enhanced insulin sensitivity and glucose metabolism via insulin receptor activation and chromodulin‐mediated signaling [8–10]. These functions support improved nutrient utilization and lipid metabolism, and Cr supplementation has been linked to improved feed conversion ratio (FCR), reduced feed intake (FI), and increased egg production (EP) [11–13]. The biological response to Cr depends not only on dietary level but also on its chemical form, with inorganic sources like chromium chloride (CrCl_3_) generally exhibiting lower bioavailability (0.5%–2%) than organic complexes (10%–25%), thereby often requiring higher inclusion levels to achieve comparable physiological responses [4, 13].

Zinc, in contrast, is an essential cofactor for numerous enzymes and transcription factors and is involved in carbohydrate metabolism, antioxidant defense, membrane stability, and insulin synthesis and signaling [6, 14]. In addition, Zn plays a critical role in eggshell formation through its involvement in carbonate deposition and structural protein synthesis [14]. Consequently, Zn supplementation has been shown to improve laying performance, feed efficiency, egg quality traits, and mineral status [15]. Because both minerals are involved in endocrine and metabolic regulation, their combined use may produce synergistic effects, although antagonistic interactions may occur due to competition in absorption and transport [6, 7, 13]. Given their limited bioavailability in plant‐based diets, Cr and Zn are commonly included in layer rations.

Despite extensive research on individual supplementation, Cr × Zn interactions remain insufficiently understood, particularly under factorial designs, with inconsistent findings reported in the literature [7, 16]. These inconsistencies likely reflect differences in dosage, mineral source, bioavailability, genetics, and physiological stage [17–19]. Moreover, most studies emphasize performance outcomes, with limited attention to integrated physiological responses such as egg quality and blood biochemical indices. Therefore, a clearer understanding of these interactions is needed to optimize trace mineral nutrition and avoid inefficiencies or adverse effects associated with excessive supplementation.

The present study evaluated the individual and interactive effects of dietary Cr and Zn supplementation on laying performance, egg quality, and selected blood biochemical parameters in Lohmann LSL‐Lite hens. It was hypothesized that Cr and Zn may exert synergistic effects through complementary metabolic pathways, while antagonistic interactions may also occur depending on dietary levels. This work aims to provide further insight into Cr–Zn interactions and support evidence‐based optimization of trace mineral nutrition in modern layer production systems.

## 2. Materials and Methods

### 2.1. Birds and General Management

**FIGURE 1:**
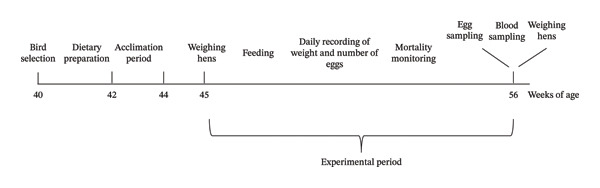
The diagram outlines the experimental timeline, which commenced with bird selection and dietary preparation (weeks 40–42), followed by an acclimation period to the new diet (weeks 42–44). Birds were weighed at the outset of the experimental period, which began at week 45. Feeding, daily recording of weight and egg numbers, and regular mortality monitoring were ongoing activities throughout the experiment (weeks 45–56). Upon completion of the experimental period (56 weeks), the chickens were weighed, and their eggs and blood were sampled for subsequent analysis.

A total of 216 Lohmann LSL‐Lite laying hens (42 weeks old; average body weight 1467 ± 140 g) were randomly assigned to six replicates of six hens each. The birds were housed in a closed, three‐tier battery cage system (45 × 45 × 45 cm; 3 hens per cage) equipped with negative‐pressure ventilation, automatic nipple drinkers, and standard feeding troughs. Two adjacent cages were considered one replicate. A light–dark cycle of 16 h light and 8 h dark was maintained, with light intensity set at 15 lux at bird level. Birds underwent a 14‐day acclimation period to the experimental diets before the start of the trial. The experiment began when birds were 42 weeks old and continued for 3 months. The housing temperature was maintained at 22 ± 2°C, with relative humidity between 30% and 40%. Each hen was provided approximately 110 g of feed daily, and water was available *ad libitum*. The experimental timeline is shown in Figure 1.

### 2.2. Experimental Design and Treatments

The experiment employed a 3 × 2 factorial design, consisting of three dietary Cr levels (0, 3, and 6 mg/kg) and two dietary Zn levels (0 and 40 mg/kg), for a total of six dietary treatments (Table 1). Zinc sulfate (ZnSO_4_, 98% purity, Merck, Germany) and chromium chloride hexahydrate (CrCl_3_·6H_2_O) served as the Zn and Cr sources, respectively. Supplements were thoroughly incorporated into the basal mash diet using a mechanical mixer to ensure uniform distribution. The basal diet was formulated to meet the nutrient requirements of Lohmann LSL‐Lite hens according to the commercial management guide and catalogue guidelines (Table 2). The sample size was determined through an a priori power analysis based on expected effect sizes reported in previous studies investigating dietary Cr and Zn supplementation in laying hens, which generally demonstrate moderate effects on performance and egg quality parameters. Assuming a significance level of *α* = 0.05 and a statistical power of 0.80 to detect both main and interaction effects within a factorial design, a minimum of six replicates per treatment was deemed adequate. Accordingly, each treatment consisted of six replicates with six hens per replicate (*n* = 36 hens per treatment). This experimental design provided sufficient statistical power to detect biologically relevant differences among treatments while maintaining consistency with established practices in poultry nutrition research.

**TABLE 1:** Experimental design and treatments.

Treatment	Cr level (mg/kg)	Zn level (mg/kg)
T1	0	0
T2	0	40
T3	3	0
T4	3	40
T5	6	0
T6	6	40

*Note:* Cr, chromium; Zn, zinc.

**TABLE 2:** Ingredients and nutritional composition of the experimental diets.

Ingredients (%)	
Corn	67.64
Wheat bran	0.15
Soybean meal	21.01
Soybean oil	0.07
Limestone	3.00
Oyster shells	5.47
Dicalcium phosphate	1.64
NaHCo3	0.18
Common salt	0.19
Vitamin premix^1^	0.25
Mineral premix^2^	0.25
DL‐Methionine	0.15

**Nutrient composition (as fed basis)**	

ME (kcal/kg)	2750
Crude protein (%)	14.69
Calcium (%)	3.64
Available phosphorus (%)	0.37
Sodium (%)	0.15
Crude fiber (%)	2.33
(Na + K)‐Cl (mEq/kg)	207
Lysine (%)	0.71
Methionine (%)	0.37
Methionine + Cystine (%)	0.63
Threonine (%)	0.54
Tryptophan (%)	0.17

^1^Per 1‐kg diet provides the following: vitamin A, 10,000 IU (all‐trans‐retinal); cholecalciferol, 2000 IU; vitamin E, 20 IU (a‐tocopheryl); vitamin K3, 3.0 mg; riboflavin, 18.0 mg; niacin, 50 mg; D‐calcium pantothenic acid, 24 mg; choline chloride, 450 mg; vitamin B12, 0.02 mg; folic acid 3.0 mg.

^2^Per 1‐kg diet provides the following: manganese, 110 mg; zinc, 50 mg; iron, 60 mg; copper, 10 mg; iodine, 100 mg; selenium, 0.2 mg.

### 2.3. Productive Performance

A 14‐day adaptation period to the experimental diets was implemented before data collection. Eggs were collected and weighed daily in the evening, and these data were used to calculate EP and egg weight (EW). The EP rate was expressed as the number of eggs produced per hen per day. Egg mass (EM) was determined as the product of EP and EW. FCR was calculated as grams of feed consumed per gram of mass produced.

To quantify FI, hens were offered approximately 110 g of feed per bird per day, and the residual feed in each feeder was recorded at the end of each week. Mortality was recorded daily, and performance parameters were adjusted accordingly. Specifically, the number of hen‐days was corrected by accounting for the exact day of mortality, and all production variables (EP, EM, FI, and FCR) were calculated using the adjusted number of live birds. This approach ensured accurate estimation of productive performance by excluding the contribution of birds that died during the experimental period.

### 2.4. Egg Quality Traits

At the end of the experiment (56 weeks old), two eggs from each replicate were randomly selected for evaluation over three consecutive days. Various egg quality traits were measured during this evaluation, including Haugh unit, egg shape index, yolk index, shell weight, shell thickness, and yolk color. Following measurement of the egg’s length and width with a compass (Swordfish, 0.02 mm, China), the egg shape index was calculated using the formula (width/length) × 100. The Haugh unit was determined using a formula [20]. The shell thickness was determined using a dial and pipe gauge (Ozaki MFG. Co., Tokyo, Japan) at three various locations on the egg (air cell, equator, and sharp end), and the mean of these three measurements was calculated to represent the average shell thickness. The yolk color was evaluated using a Roche fan scale (DSM, Switzerland). Yolk index was calculated for each egg using the formula, yolk index = (H/D) × 100, where H represents yolk height and D represents yolk diameter. H and D were measured using a tripod micrometer (Mitutoyo, 0.01 mm, Japan) and a compass (Swordfish, 0.02 mm, China), respectively.

### 2.5. Blood Parameters

At the end of the experiment (56 weeks of age), one hen was randomly selected from each group. Blood samples (3 mL) were collected from the brachial (wing) vein into test tubes with heparin and without anticoagulant. The samples were centrifuged at 3000 rpm for 15 min, and the resulting plasma and serum were stored at −20°C until laboratory analysis. Albumin, glucose, total triglyceride, total cholesterol, and uric acid concentrations were determined according to the manufacturer’s instructions using Pars Azmun diagnostic kits (Tehran, Iran). Plasma Cr and Zn concentrations were measured using an atomic absorption spectrophotometer (Perkin Elmer HGA 500).

### 2.6. Statistical Analysis

The data were analyzed using analysis of variance (ANOVA) in a completely randomized design (CRD) with a 3 × 2 factorial arrangement of treatments. The analysis was conducted using the general linear model (GLM) procedure in SAS 9.4 software [21]. The following model was applied to analyze the data:

(1) Yijk=μ+Ai+Bj+ABij+eijk.

In this model, *Y*_*i* *j* *k*_ represents the observed value for a specific trait, *μ* represents the overall mean (the average value of the trait across all observations), *A*_*i*_ represents the main effect of Zn, *B*_*j*_ represents the main effect of Cr, *A* *B*_*i* *j*_ represents the respective interaction of *i*th and *j*th levels of dietary Zn and Cr, and *e*_*i* *j* *k*_ represents random error associated with the *i* *j* *k*_*t* *h*_ recording. Significance was declared at *p* ≤ 0.05, and treatment means were compared using Tukey’s honestly significant difference (HSD) test. When the interaction between Cr and Zn was significant, simple effects were examined and results were interpreted based on the combined treatment means. In the absence of a significant interaction, the main effects of Cr and Zn were interpreted independently. The experimental unit for performance parameters (e.g., FI, EP, EM, and FCR) was the replicate cage, whereas for egg quality traits and blood biochemical parameters, the individual bird was considered the experimental unit. To complement hypothesis testing and quantify treatment effects, partial eta‐squared (*η* ^2^p) was calculated for the main effects of Zn and Cr, as well as for their interaction, using the following formula:

(2) η2p=SSeffectSSeffect+SSerror

where SS_effect_ is the sum of squares for the specific factor and SS_effect_ is the error sum of squares. Effect sizes were interpreted according to Cohen [22], where 0.01, 0.06, and 0.14 represent small, medium, and large effects, respectively.

## 3. Results

### 3.1. Productive Performance

The effects of dietary Cr and Zn supplementation on productive performance are presented in Tables 3 and 4. No significant Zn × Cr interactions were observed for any of the measured parameters (*p* > 0.05). FI was not significantly affected by either Zn or Cr supplementation across all experimental periods (*p* > 0.05), although Cr showed moderate effect sizes for FI variables (*η* ^2^ *p* = 0.103–0.157). Chromium supplementation significantly impaired FCR, particularly in Months 1, 2, and over the entire period (*p* = 0.001, *p* = 0.050, and *p* = 0.001, respectively). Hens receiving 0 mg/kg Cr exhibited lower FCR values during Month 1 and across Months 1–3 compared with those supplemented with 3 or 6 mg/kg Cr. Partial eta‐squared values indicated a large effect size of Cr on FCR, particularly during Month 1 (*η* ^2^ *p* = 0.787) and over the entire experimental period (*η* ^2^ *p* = 0.497). EP was also significantly reduced by Cr supplementation (*p* = 0.001). In Month 1, EP declined from 87.86% in the control group to 69.18% and 66.22% at 3 and 6 mg/kg Cr. Over the entire period, EP decreased from 88.92% to 84.01% and 80.00%. The effect size analysis revealed a very large effect of Cr on EP during Month 1 (*η* ^2^ *p* = 0.859) and a moderate‐to‐large effect over the entire experimental period (*η* ^2^ *p* = 0.447). Similarly, EM was significantly affected by Cr supplementation during Month 3 (*p* = 0.001) and across the overall experimental period (*p* = 0.043). Birds fed 0 mg/kg Cr exhibited greater EM values during Month 3 and throughout Months 1–3 compared with those receiving 3 or 6 mg/kg Cr. Partial eta‐squared values indicated a large effect of Cr on EM in Month 1 (*η* ^2^ *p* = 0.853) and a moderate‐to‐large overall effect across the experimental period (*η* ^2^ *p* = 0.453). In contrast, dietary Zn supplementation had no significant effect on FI, FCR, EP, or EM throughout the experimental period (*p* > 0.05).

**TABLE 3:** Influence of dietary Cr and Zn on feed intake and feed conversion ratio of laying hens.

Interactions		Feed intake (g/hen/d)				Feed conversion ratio (g feed: g egg)			
		Month 1	Month 2	Month 3	Month 1–3	Month 1	Month 2	Month 3	Month 1–3
*Zn (mg/kg)*	*Cr (mg/kg)*								
0	0	109.49	109.77	108.37	109.21	1.97	1.89	1.89	1.92
0	3	108.62	109.42	105.69	107.91	2.52	1.96	1.82	2.10
0	6	109.22	109.40	108.95	109.19	2.70	1.99	1.96	2.22
40	0	109.71	109.90	107.48	109.03	1.95	1.88	1.82	1.89
40	3	108.68	110.00	106.88	108.52	2.44	1.80	1.81	2.02
40	6	108.76	108.96	107.54	108.42	2.53	2.03	1.90	2.15

*Zn (mg/kg)*									
0		109.11	109.53	107.67	108.77	2.39	1.94	1.89	2.07
40		109.05	109.62	107.29	108.65	2.30	1.90	1.84	2.02

*Cr (mg/kg)*									
0		109.60	109.84	107.92	109.12	1.96^b^	1.89^b^	1.85	1.90^b^
3		108.65	109.70	106.28	108.21	2.48^a^	1.88^b^	1.81	2.06^a^
6		108.99	109.18	108.24	108.80	2.61^a^	2.01^a^	1.92	2.18^a^

*p* *value*									
Zn		0.859	0.743	0.692	0.770	0.107	0.399	0.278	0.179
Cr		0.076	0.126	0.197	0.168	0.001	0.050	0.122	0.001
Zn × Cr		0.682	0.316	0.489	0.358	0.460	0.255	0.865	0.875

*F-value*									
Zn		0.03	0.11	0.16	0.09	2.76	0.73	1.22	1.89
Cr		2.80	2.22	1.71	1.89	55.37	3.16	2.25	14.84
Zn × Cr		0.39	1.20	0.73	1.06	0.80	1.43	0.14	0.13

*DF*									
Zn		1	1	1	1	1	1	1	1
Cr		2	2	2	2	2	2	2	2
Zn × Cr		2	2	2	2	2	2	2	2

*η* ^2^ *p*									
Zn		0.001	0.003	0.005	0.003	0.084	0.024	0.039	0.059
Cr		0.157	0.129	0.103	0.112	0.787	0.174	0.131	0.497
Zn × Cr		0.025	0.074	0.047	0.066	0.050	0.087	0.100	0.009

*Note:* The presence of different superscript letters within a column indicates statistically significant differences (*p* ≤ 0.05) between the corresponding means (*n* = 6 replicates/treatment, 6 animals/replicate). Cr, chromium; Zn, zinc; *η* ^2^p = partial eta‐squared (effect size).

Abbreviation: DF, degrees of freedom.

**TABLE 4:** Influence of dietary Cr and Zn on egg production and egg mass of laying hens.

Interactions		Egg production (%)				Egg mass (g/hen/d)			
		Month 1	Month 2	Month 3	Month 1–3	Month 1	Month 2	Month 3	Month 1–3
*Zn (mg/kg)*	*Cr (mg/kg)*								
0	0	87.53	89.66	88.02	88.40	58.25	57.65	55.95	57.28
0	3	68.65	87.77	91.67	82.70	56.32	58.08	43.07	52.49
0	6	63.79	86.01	85.96	78.59	55.39	56.02	40.82	50.75
40	0	88.20	89.78	90.38	89.45	58.30	59.05	56.03	57.79
40	3	69.72	94.04	92.22	85.33	60.96	59.20	44.60	54.92
40	6	68.65	85.51	90.08	81.41	53.87	56.80	43.02	51.23

*Zn (mg/kg)*									
0		73.32	87.81	88.54	83.22	56.08	57.25	46.61	53.31
40		75.52	89.78	90.89	85.39	52.98	58.35	47.88	53.07
*Cr (mg/kg)*									
0		87.86^a^	89.72	89.19	88.92^a^	53.56	58.35	55.98^a^	55.96^a^
3		69.18^b^	90.90	91.94	84.01^b^	52.73	58.35	43.83^b^	51.74^b^
6		66.22^b^	85.76	88.02	80.00^b^	57.30	56.41	41.92^b^	51.88^b^

*p* *value*									
Zn		0.131	0.337	0.162	0.154	0.324	0.377	0.189	0.869
Cr		0.001	0.110	0.150	0.001	0.449	0.287	0.001	0.043
Zn × Cr		0.422	0.331	0.677	0.866	0.337	0.979	0.648	0.520

*F-value*									
Zn		2.40	0.95	2.05	2.14	1.81	0.55	0.80	1.12
Cr		91.14	2.37	2.02	12.10	87.01	3.23	1.30	12.40
Zn × Cr		0.89	1.15	0.39	0.14	0.44	1.68	0.02	0.36

*DF*									
Zn		1	1	1	1	1	1	1	1
Cr		2	2	2	2	2	2	2	2
Zn × Cr		2	2	2	2	2	2	2	2

*η* ^2^ *p*									
Zn		0.074	0.031	0.064	0.067	0.057	0.018	0.026	0.036
Cr		0.859	0.137	0.118	0.447	0.853	0.177	0.080	0.453
Zn × Cr		0.056	0.071	0.026	0.010	0.028	0.101	0.001	0.023

*Note:* The presence of different superscript letters within a column indicates statistically significant differences (*p* ≤ 0.05) between the corresponding means (*n* = 6 replicates/treatment, 6 animals/replicate). Cr, chromium; Zn, zinc; *η* ^2^p = partial eta‐squared (effect size).

Abbreviation: DF, degrees of freedom.

### 3.2. Egg Quality Traits

Table 5 presents the effects of dietary Cr and Zn supplementation on egg quality traits in laying hens. Neither Cr nor Zn supplementation significantly affected EW, egg index, yolk index, or Haugh unit (*p* > 0.05). However, a significant Cr × Zn interaction was observed for yolk color (*p* = 0.0429). Hens receiving 40 mg/kg Zn in combination with 3 mg/kg Cr exhibited a higher yolk color score compared with both the control group and the group supplemented with 40 mg/kg Zn plus 6 mg/kg Cr, whereas the remaining treatment groups did not differ significantly from the control (*p* > 0.05). The interaction effect size for yolk color was moderate (*η* ^2^ *p* = 0.191). Dietary Cr supplementation significantly affected shell weight (*p* = 0.0138), with hens fed 3 or 6 mg/kg Cr exhibiting lower shell weights than those receiving no supplemental Cr. In addition, both Cr and Zn significantly influenced shell thickness (*p* = 0.0259 and *p* = 0.0026, respectively). Supplementation with 3 or 6 mg/kg Cr reduced shell thickness, whereas dietary inclusion of 40 mg/kg Zn increased shell thickness relative to the unsupplemented control. Effect size analysis indicated moderate effects of Cr on shell weight (*η* ^2^ *p* = 0.250) and shell thickness (*η* ^2^ *p* = 0.260).

**TABLE 5:** Influence of dietary Cr and Zn on laying hen egg quality.

Interactions		Egg weight (g)	Egg index	Yolk index	Haugh unit	Yolk color (Roche)	Shell weight (g)	Shell thickness (mm10^−2^)
*Zn (mg/kg)*	*Cr (mg/kg)*							
0	0	64.65	72.94	39.10	77.84	6.55^b^	6.09	38.83
0	3	65.68	74.26	39.61	76.13	6.72^ab^	5.93	37.16
0	6	63.65	75.00	38.18	80.78	6.77^ab^	5.73	37.50
40	0	65.04	74.15	40.29	84.60	6.78^ab^	6.26	40.27
40	3	65.03	74.34	39.60	77.27	6.94^a^	6.00	39.00
40	6	64.84	73.67	39.32	79.54	6.55^b^	5.74	38.83

*Zn (mg/kg)*								
0		64.66	74.08	38.96	78.25	6.68	5.92	37.83^b^
40		64.97	74.06	39.75	80.47	6.76	6.01	39.37^a^

*Cr (mg/kg)*								
0		64.85	73.57	39.69	81.22	6.67	6.18^a^	39.55^a^
3		65.36	74.30	39.60	76.70	6.83	5.96^b^	38.08^b^
6		64.25	74.34	38.76	80.17	6.67	5.74^b^	38.17^b^

*p* *value*								
Zn		0.7710	0.9631	0.1672	0.3903	0.3489	0.4546	0.0026
Cr		0.6910	0.5283	0.3083	0.3306	0.1546	0.0138	0.0259
Zn × Cr		0.7744	0.2694	0.6121	0.4307	0.0429	0.8335	0.9014

*F-value*								
Zn		0.49	0.00	0.32	1.81	0.22	1.48	7.73
Cr		0.22	1.02	0.16	1.35	3.10	4.02	4.23
Zn × Cr		0.21	1.00	1.08	0.14	2.73	0.67	1.30

*DF*								
Zn		1	1	1	1	1	1	1
Cr		2	2	2	2	2	2	2
Zn × Cr		2	2	2	2	2	2	2

*η* ^2^ *p*								
Zn		0.003	0.011	0.006	0.025	0.030	0.020	0.022
Cr		0.024	0.040	0.034	0.071	0.120	0.250	0.260
Zn × Cr		0.017	0.080	0.028	0.055	0.191	0.010	0.010

*Note:* The presence of different superscript letters within a column indicates statistically significant differences (*p* ≤ 0.05) between the corresponding means (*n* = 6 replicates/treatment (2 eggs/replicate, measured over 3 days)). Cr, chromium; Zn, zinc; *η* ^2^p = partial eta‐squared (effect size).

Abbreviation: DF, degrees of freedom.

### 3.3. Blood Parameters

Table 6 presents the effects of dietary Cr and Zn supplementation on blood biochemical parameters of laying hens. Serum uric acid, glucose, and cholesterol concentrations were not affected by dietary treatments (*p* > 0.05). Significant main effects of Cr and Zn were observed on serum albumin concentration (*p* = 0.0001 for both). Hens fed diets supplemented with 6 mg/kg Cr and 40 mg/kg Zn exhibited significantly higher albumin levels compared with the control group. Partial eta‐squared values indicated a large effect of Cr on albumin (*η* ^2^ *p* = 0.461), while Zn showed a moderate‐to‐large effect (*η* ^2^ *p* = 0.401). Serum triglyceride concentration was significantly influenced by Zn supplementation (*p* = 0.0490). Birds receiving 40 mg/kg Zn had lower triglyceride levels compared with the control group (*p* < 0.05). The effect size of Zn on triglycerides was small to moderate (*η* ^2^ *p* = 0.121). Zinc supplementation also significantly affected serum Zn concentration (*p* = 0.0267), with the 40 mg/kg Zn treatment resulting in higher values than the control diet. The effect size was moderate (*η* ^2^ *p* = 0.153). A significant interaction between dietary Cr and Zn was observed for serum Cr concentration (*p* = 0.0001). The control group showed the highest Cr concentrations, which were significantly greater than all other treatments (Table 6). The interaction effect size was large (*η* ^2^ *p* = 0.607).

**TABLE 6:** Influence of dietary Cr and Zn on blood parameters of laying hens.

Interaction		Albumin (g/dL)	Uric acid (mg/dL)	Glucose (mg/dL)	Triglyceride (mg/dL)	Cholesterol (mg/dL)	Zn (mg/dL)	Cr (mg/dL)
*Zn (mg/kg)*	*Cr (mg/kg)*							
0	0	3.87	7.48	145.61	701.02	1247.60	0.37	0.48^a^
0	3	4.47	8.18	139.92	768.92	1229.12	0.37	0.13^b^
0	6	6.98	9.38	133.98	644.79	1161.41	0.44	0.11^b^
40	0	5.95	8.17	133.75	582.69	1097.91	0.49	0.09^b^
40	3	7.13	8.66	135.94	664.82	1169.76	0.42	0.11^b^
40	6	8.35	9.17	143.48	592.06	1216.56	0.47	0.09^b^

*Zn (mg/kg)*								
0		5.11^b^	8.35	139.83	704.91^a^	1212.71	0.40^b^	0.24^a^
40		7.14^a^	8.67	137.72	613.19^b^	1161.41	0.46^a^	0.11^b^

*Cr (mg/kg)*								
0		4.91^b^	7.82	139.68	641.85	1172.76	0.43	0.28^a^
3		5.80^b^	8.42	137.93	716.87	1199.44	0.40	0.12^b^
6		7.67^a^	9.27	138.73	618.43	1188.98	0.46	0.10^b^

*p* *value*								
Zn		0.0001	0.6643	0.7775	0.0490	0.4997	0.0267	0.0001
Cr		0.0001	0.2819	0.9817	0.1938	0.9582	0.2021	0.0001
Zn × Cr		0.5176	0.8709	0.5011	0.8237	0.5431	0.3739	0.0001

*F-value*								
Zn		20.12	0.19	0.08	5.15	0.47	5.43	31.68
Cr		12.83	1.32	0.02	1.73	0.04	1.69	20.75
Zn × Cr		0.67	0.14	0.71	0.20	0.62	1.02	23.14

*DF*								
Zn		1	1	1	1	1	1	1
Cr		2	2	2	2	2	2	2
Zn × Cr		2	2	2	2	2	2	2

*η* ^2^ *p*								
Zn		0.401	0.006	0.003	0.121	0.015	0.153	0.514
Cr		0.461	0.081	0.001	0.104	0.003	0.101	0.580
Zn × Cr		0.043	0.009	0.045	0.013	0.040	0.063	0.607

*Note:* The presence of different superscript letters within a column indicates statistically significant differences (*p* ≤ 0.05) between the corresponding means (*n* = 6 replicates/treatment, 1 animal/replicate). Cr, chromium; Zn, zinc; *η* ^2^p = partial eta‐squared (effect size).

Abbreviation: DF, degrees of freedom.

## 4. Discussion

### 4.1. Productive Performance

In the present study, supplemental Zn (40 mg/kg) did not influence laying performance, whereas Cr markedly impaired efficiency and egg output. The absence of a Zn response likely indicates that basal dietary levels were sufficient to meet physiological requirements (≈35 mg/kg), thereby limiting the marginal benefit of additional supplementation [14]. This lack of effect may further reflect the use of inorganic Zn sources with lower bioavailability compared to organic forms, as well as potential strain‐specific differences in mineral requirements in modern high‐producing layers [14, 15]. Indeed, while several studies have reported that Zn, especially in organic forms, enhances laying rate and feed efficiency [15, 23], others have observed no significant performance improvements [19, 24–26], highlighting the importance of mineral form, dietary adequacy, and bird physiology in determining outcomes.

In contrast, Cr supplementation (3 and 6 mg/kg as CrCl_3_·6H_2_O) significantly increased FCR and reduced EP and EM, without affecting FI. This indicates that Cr impaired nutrient utilization efficiency rather than appetite. Similar observations of unchanged FI have been reported in previous studies evaluating Cr or Zn supplementation [15, 27, 28], although reductions in intake have also been documented depending on Cr source and dose [12, 29].

Over the 12‐week period, Cr supplementation increased FCR by approximately 8%–15% and reduced EP by 6%–10%, demonstrating a clear negative effect on production efficiency. These findings contrast with studies reporting beneficial effects of low‐dose, organic Cr supplementation under stress conditions. For instance, Sahin et al. [30] and Sahin et al. [31] reported improved EP and feed efficiency in hens and quail and exposed to cold and heat stress, respectively, when supplemented with ≤ 1.2 mg/kg Cr as Cr picolinate. These improvements are generally attributed to enhanced insulin sensitivity and reduced stress hormone levels, which promote nutrient utilization [6, 13]. However, such responses appear to be context‐dependent and dose‐sensitive. At higher inclusion levels, particularly in inorganic forms, Cr may exert neutral or adverse effects. Previous studies have reported no improvement or inconsistent responses in EP and EM at elevated Cr levels [12, 27, 32, 33], supporting the notion that excessive Cr can overwhelm normal metabolic regulation.

The deleterious effects observed in the present study may be explained by several interrelated mechanisms. First, excessive Cr may disrupt glucose–insulin homeostasis. Although Cr enhances insulin action at physiological levels, supranutritional doses may dysregulate glucose metabolism, potentially inducing hypoglycemia or altering nutrient partitioning, thereby limiting energy availability for egg formation and increasing FCR [8, 9, 34]. Supporting this, Uyanik et al. [12] reported reduced serum glucose and altered mineral profiles in broilers receiving high CrCl_3_. Second, high Cr levels may induce oxidative stress. Even trivalent Cr, when present in excess, can promote reactive oxygen species (ROS) formation, leading to cellular damage in hepatic and reproductive tissues [35]. Such oxidative stress may impair nutrient utilization and steroidogenesis, ultimately reducing EM and production efficiency [35]. Third, the low bioavailability of inorganic CrCl_3_ suggests that a substantial proportion of supplemented Cr remained unabsorbed in the gastrointestinal tract. This may interfere with the absorption of other essential minerals, such as Zn and Cu, or disrupt gut integrity and function, indirectly compromising productivity [13, 32].

No significant Cr × Zn interactions were detected for any performance parameters. This contrasts with reports of synergistic effects under stress conditions, where combined supplementation improved performance traits [16]. The absence of interaction in the present study may reflect the high Cr inclusion levels, potential competitive interactions at the level of intestinal absorption, or the lack of environmental or physiological stressors necessary to elicit synergistic responses.

### 4.2. Egg Quality Traits

This study found no significant effects of dietary treatments on the Haugh unit, egg index, or yolk index. This aligns with previous research where Cr supplementation had no impact on these parameters [12, 33, 36]. Similarly, no significant differences in egg quality parameters were observed in hens fed varying levels of Zn [25].

In the present study, the combination of 40 mg/kg Zn and 3 mg/kg Cr resulted in the most intense yolk pigmentation, whereas both the control and the higher Cr level (40 mg/kg Zn + 6 mg/kg Cr) produced the palest yolks. Yolk color is a key quality attribute influencing consumer preference and market value, largely determined by the absorption, transport, and deposition of dietary carotenoids into the yolk [37]. These processes depend on efficient lipid digestion and very‐low‐density lipoprotein (VLDL) metabolism [37, 38].

Zinc likely contributed to enhanced pigmentation through its role in maintaining intestinal integrity and supporting lipid digestion and transport [39]. As a cofactor for enzymes involved in bile secretion and lipoprotein synthesis, Zn facilitates micellar solubilization and subsequent absorption of fat‐soluble carotenoids [39]. Accordingly, adequate Zn status may promote efficient carotenoid uptake and deposition, whereas deficiency has been associated with reduced pigmentation [39, 40].

In contrast, the role of Cr in yolk pigmentation remains unclear and appears dose‐dependent. Previous studies have reported inconsistent effects, ranging from no change to reduced yolk color depending on Cr source and inclusion level [19, 33, 36]. The present results suggest that moderate Cr supplementation (3 mg/kg) may have supported carotenoid deposition, possibly via improved metabolic efficiency, whereas higher levels (6 mg/kg) may have disrupted lipid metabolism or pigment stability, thereby attenuating the positive effect of Zn. This pattern highlights a potential threshold beyond which Cr exerts antagonistic rather than supportive effects on yolk pigmentation.

In the present study, dietary supplementation with 40 mg/kg Zn significantly increased eggshell thickness without affecting shell weight, corroborating earlier findings in laying hens [16, 41]. The most plausible mechanism is Zn’s essential role as a cofactor for carbonic anhydrase, the shell gland enzyme responsible for catalyzing the hydration of CO_2_ to bicarbonate, thereby providing carbonate ions for CaCO_3_ deposition [14]. Enhanced carbonic anhydrase activity can facilitate a greater flux of carbonate to the mineralization front, resulting in denser shell deposition. Zn may also improve calcium (Ca) utilization efficiency [14, 42], further supporting shell formation and structural integrity.

In contrast, high dietary Cr (6 mg/kg) reduced both shell thickness and shell weight in our hens. This negative effect contrasts with reports where Cr supplementation, particularly under heat stress, improved shell quality [43]. A plausible explanation is that excessive Cr disrupts mineral metabolism or interferes with endocrine regulation of shell gland function, potentially via altered Ca homeostasis or feedback inhibition of vitellogenic processes [44, 45]. The literature on Cr’s influence on shell quality is inconsistent: several studies found no significant effects [12, 29, 36], whereas others reported both detrimental and beneficial responses depending on dose and source. For example, Lien et al. [46] observed reduced shell thickness with 400 μg/kg Cr as Cr picolinate, whereas Ma et al. [33] reported increased thickness at 600 μg/kg Cr as Cr propionate. Such variability likely reflects differences in Cr chemical form, inclusion level, bird physiological status, and environmental conditions. The interplay between Cr, Zn, and the mineral matrix of the eggshell further complicates interpretation. Ca is the principal mineral in the eggshell, while phosphorus (P) plays a supportive role in shell matrix formation [47]. Cr’s influence on mineral metabolism in layers is unclear—Uyanik et al. [12] found elevated serum Ca without changes in P after 20 ppm CrCl_3_·6H_2_O, while Page et al. [48] and Sahin et al. [49] reported no effect on serum Ca or inorganic P. According to Elnesr et al. [14], the benefits of Cr on shell quality may be more pronounced at non‐thermoneutral temperatures, suggesting that environmental stress modulates Cr’s efficacy.

### 4.3. Blood Parameters

Serum uric acid, glucose, and cholesterol levels were not affected by the treatments, indicating that protein catabolism and energy metabolism remained stable. This is consistent with previous research where no significant impacts in these levels were observed in laying hens supplemented with varying amounts of Cr propionate (200, 400, or 600 μg/kg) [33]. However, a different study reported a decrease in blood sugar following Cr propionate supplementation [10], which could be attributed to differences in the baseline Cr levels of the hens or variations in the experimental protocols, such as the source of Cr or the duration of supplementation. Likewise, another study found that adding different forms of Zn to the diet did not influence blood glucose levels compared to the control group [50].

Our study showed that increasing dietary Cr and Zn levels resulted in higher blood albumin concentrations in laying hens. This corroborates Karami et al. [19], who reported higher albumin concentrations following Zn supplementation. Albumin is a multifunctional plasma protein that maintains oncotic pressure, transports hormones, fatty acids, and minerals, and binds approximately 80% of circulating Zn [51]. Its elevation may reflect improved protein nutritional status or enhanced hepatic synthetic capacity. While Ma et al. [33] and Sedgh‐Gooya and Torki [52] found no direct effect of Cr on albumin levels, Cr may indirectly influence albumin production through improved insulin sensitivity [8], or enhanced amino acid uptake and protein synthesis [53]. Such metabolic effects could plausibly contribute to the modest increases observed in our hens.

Dietary Zn at 40 mg/kg significantly reduced serum triglycerides, whereas Cr supplementation alone did not alter triglycerides or cholesterol. These results are consistent with multiple studies reporting no Cr effect on serum lipids in laying hens [12, 27, 33, 34], but align with evidence that Zn supplementation decreases triglycerides [54, 55]. The lipid‐lowering effect of Zn may involve enhanced insulin secretion and storage, upregulation of lipoprotein lipase activity, and/or suppression of hepatic lipogenesis [39], ultimately reducing circulating triglycerides. Additionally, Zn may promote lactic acid bacterial growth in the gut [56], which can reduce cholesterol via bile salt deconjugation and increased sterol excretion.

Adding Zn to the diet of laying hens increased blood serum Zn concentration. This is consistent with previous research where Zn supplementation in laying hen diets resulted in elevated blood serum Zn levels [15, 55, 57]. These findings indicate a positive correlation between dietary Zn intake and its presence in the bloodstream.

The interaction between Zn and Cr on blood Cr levels, however, appears more complex. While some studies report that Cr supplementation alone increases serum Cr [19, 58], the present study observed a decrease in blood Cr concentration when both Zn and Cr were elevated in the diet. This decrease should not be interpreted as reduced absorption or bioavailability, since circulating trace minerals are tightly regulated by homeostasis, including tissue uptake and excretion [59, 60]. It is likely that supplemented Cr was rapidly redistributed to metabolically active tissues or utilized in insulin‐related pathways, although this remains speculative due to the lack of tissue measurements. Thus, the results more likely reflect altered Cr distribution rather than reduced availability.

Antagonistic interactions between Zn and Cr may also explain this outcome. Both minerals may compete for intestinal absorption pathways, with Zn potentially limiting Cr uptake [6, 60]. Additionally, Zn can induce metallothionein production, promoting Cr sequestration in tissues and lowering circulating levels [15]. The inherently low bioavailability of Cr, especially in inorganic forms, further contributes, as higher dietary levels often result in reduced absorption and increased excretion [4]. Renal elimination and indirect metabolic effects may also play roles in maintaining Cr homeostasis [61].

## 5. Conclusions

This study investigated the effects of dietary Cr and Zn supplementation on laying hen performance, egg quality, and blood biochemical parameters. The results demonstrated that FI was not affected by either mineral; however, Cr supplementation at 3 and 6 mg/kg impaired FCR and reduced EP and EM, particularly during the later stages of the trial. Although Zn supplementation did not significantly affect overall productive performance, a significant Cr × Zn interaction improved yolk color, with the highest values observed in hens receiving 3 mg/kg Cr combined with 40 mg/kg Zn. Chromium supplementation reduced eggshell weight and thickness irrespective of dose, whereas Zn supplementation at 40 mg/kg enhanced shell thickness. Blood biochemical analyses revealed that increasing dietary Cr and Zn levels elevated albumin concentrations. In addition, Zn supplementation reduced triglyceride levels and increased blood Zn concentrations, whereas Cr supplementation decreased blood Cr concentrations relative to the control group.

From a practical standpoint, Zn supplementation at 40 mg/kg may be recommended to improve eggshell quality and certain blood biochemical parameters in laying hens. In contrast, Cr supplementation at the tested levels (3 and 6 mg/kg), particularly in inorganic form, did not provide performance benefits and may negatively affect productivity and eggshell quality. Therefore, routine dietary inclusion of Cr at these levels is not recommended under similar production conditions, and caution should be exercised to avoid unnecessary or excessive supplementation.

This study has several limitations. Only two Zn supplementation levels and three Cr levels were evaluated, which may not fully represent the dose–response relationship between these minerals and laying hen performance. Furthermore, the experimental period was limited to 12 weeks and involved only a single laying hen strain, which may limit the generalizability of the findings.

Future studies should evaluate a broader range of supplementation levels, longer‐term effects, and the mechanisms underlying Cr and Zn interactions. Research across different genetic lines and environmental conditions would further support more precise mineral nutrition strategies in poultry production. In addition, more extensive within‐replicate sampling of egg quality and blood parameters is recommended to better capture variability and strengthen result robustness.

## Author Contributions

Shadi Sedgh‐Gooya: data curation, formal analysis, methodology, software, visualization, and writing–original draft. Ahmad Mohebbifar: conceptualization, data curation, formal analysis, investigation, methodology, and project administration. Mehran Torki: conceptualization, funding acquisition, project administration, resources, software, supervision, validation, and Writing–review and editing.

## Funding

The preparation of this manuscript did not receive financial assistance.

## Ethics Statement

The Animal Ethics Committee of Razi University, Kermanshah, Iran, provided approval for the experimental protocols, which followed the EU standards for animal protection and/or feed legislation.

## Conflicts of Interest

The authors declare no conflicts of interest.

## Data Availability

Data will be made available upon request.

## References

[1] Nys Y. , Schlegel P. , Durosoy S. , Jondreville C. , and Narcy A. , Adapting Trace Mineral Nutrition of Birds for Optimising the Environment and Poultry Product Quality, World’s Poultry Science Journal. (2018) 74, no. 2, 225–238, 10.1017/S0043933918000016.

[2] Richards J. D. , Zhao J. , Harrell R. J. , Atwell C. A. , and Dibner J. J. , Trace Mineral Nutrition in Poultry and Swine, Asian-Australasian Journal of Animal Sciences. (2010) 23, no. 11, 1527–1534, 10.5713/ajas.2010.r.07.

[3] NRC , Nutrient Requirements of Poultry, 1994, NRC.

[4] Haq Z. , Jain R. K. , Khan N. et al., Recent Advances in Role of Chromium and Its Antioxidant Combinations in Poultry Nutrition: A Review, Veterinary World. (2016) 9, no. 12, 1392–1399, 10.14202/vetworld.2016.1392-1399. 28096611 PMC5234053

[5] Underwood G. , Andrews D. , and Phung T. , Advances in Genetic Selection and Breeder Practice Improve Commercial Layer Hen Welfare, Animal Production Science. (2021) 61, no. 10, 856–866, 10.1071/AN20383.

[6] Jawad Musafer K. N. , Zulkeflee H. A. , Wan N. W. N. F. H. , Ab Rahim S. N. , and Tuan Ismail T. S. , Unraveling the Complex Role of Zinc, Boron, Chromium, and Selenium in the Pathogenesis of Diabetes Mellitus: A Review, International Islamic University Malaysia Medical Journal. (2024) 23, 10.31436/imjm.v23i03.2459.

[7] Mohamed A. S. , Abd El Latif M. A. , Hussein E. A. et al., Efficacy of Dietary Supplementation with zinc-chromium Mixture, Organic Selenium, or Their Combinations on Growth Performance, Carcass Traits, and Blood Profiles of Broilers Under Heat Stress Conditions, Animals. (2023) 13, no. 15, 10.3390/ani13152539. PMC10416910 37570347

[8] Hua Y. , Clark S. , Ren J. , and Sreejayan N. , Molecular Mechanisms of Chromium in Alleviating Insulin Resistance, The Journal of Nutritional Biochemistry. (2012) 23, no. 4, 313–319, 10.1016/j.jnutbio.2011.11.001. 22423897 PMC3308119

[9] White P. E. and Vincent B. , Systematic Review of the Effects of Chromium (III) on Chickens, Biological Trace Element Research. (2019) 188, no. 1, 99–126, 10.1007/s12011-018-1575-8. 30430417

[10] Arif M. , Hussain I. , Mahmood M. A. et al., Effect of Varying Levels of Chromium Propionate on Growth Performance and Blood Biochemistry of Broilers, Animals. (2019) 9, no. 11, 10.3390/ani9110935. PMC6912386 31703417

[11] Şahin K. , Küçük O. S. , Şahin N. , and Ozbey O. , Effects of Dietary Chromium Picolinate Supplementation on Egg Production, Egg Quality and Serum Concentrations of Insulin, Corticosterone, and Some Metabolites of Japanese Quails, Nutrition Research. (2001) 21, no. 9, 1315–1321, 10.1016/S0271-5317(01)00330-X.

[12] Uyanik F. , Kaya S. , Kolsuz A. H. , Eren M. , and Sahin N. , The Effect of Cr Supplementation on Egg Production, Egg Quality and Some Serum Parameters in Laying Hens, Turkish Journal of Veterinary and Animal Sciences. (2002) 26, 379–387.

[13] Haloi S. , Srihitha S. , and Dixit C. P. , Chromium and Its Role in Poultry Nutrition, Journal of Livestock Science. (2025) 16, 144–151, 10.33259/JLivestSci.2024.144-151.

[14] Elnesr S. S. , Mahmoud B. Y. , da Silva Pires P. G. et al., Trace Minerals in Laying Hen Diets and Their Effects on Egg Quality, Biological Trace Element Research. (2024) 202, no. 12, 5664–5679, 10.1007/s12011-024-04121-8. 38424327 PMC11502586

[15] Ogbuewu I. P. and Mbajiorgu C. A. , Meta-Analysis of Zinc Supplementation on Laying Performance, Egg Quality Characteristics, and Blood Zinc Concentrations in Laying Hens, Biological Trace Element Research. (2022) 200, no. 12, 5188–5204, 10.1007/s12011-021-03080-8. 35112232

[16] Sahin N. , Onderci M. , and Sahin K. , Effects of Dietary Chromium and Zinc on Egg Production, Egg Quality, and Some Blood Metabolites of Laying Hens Reared Under Low Ambient Temperature, Biological Trace Element Research. (2002) 85, no. 1, 47–58, 10.1385/BTER:85:1:47. 11881798

[17] Onderci M. , Sahin N. , Sahin K. , and Kilic N. , Antioxidant Properties of Chromium and Zinc: In Vivo Effects on Digestibility, Lipid Peroxidation, Antioxidant Vitamins, and Some Minerals Under a Low Ambient Temperature, Biological Trace Element Research. (2003) 92, no. 2, 139–149, 10.1385/BTER:92:2:139. 12746573

[18] Bao Y. M. , Choct M. , Iji P. A. , and Bruerton K. , Trace Mineral Interactions in Broiler Chicken Diets, British Poultry Science. (2010) 51, no. 1, 109–117, 10.1080/00071660903571904. 20390575

[19] Karami M. , Torki M. , and Mohammadi H. , Effects of Dietary Supplemental Chromium Methionine, Zinc Oxide, and Ascorbic Acid on Performance, Egg Quality Traits, and Blood Parameters of Laying Hens Subjected to Heat Stress, Journal of Applied Animal Research. (2018) 46, no. 1, 1174–1184, 10.1080/09712119.2018.1481411.

[20] Eisen E. J. , Bohren B. B. , and McKean H. E. , The Haugh Unit as a Measure of Egg Albumen Quality, Poultry Science. (1962) 41, no. 5, 1461–1468, 10.3382/ps.0411461.

[21] Statistical Analyses System (SAS) , SAS User’s Guide, Version 9.4, 2015, SAS Inst.

[22] Cohen J. , Statistical Power Analysis for the Behavioral Sciences, 2013, Routledge.

[23] Niknia A. D. , Vakili R. , and Tahmasbi A. M. , Zinc Supplementation Improves Antioxidant Status, and Organic Zinc is More Efficient Than Inorganic Zinc in Improving the Bone Strength of Aged Laying Hens, Veterinary Medical Science. (2022) 8, no. 5, 2040–2049, 10.1002/vms3.896. PMC9514485 35925611

[24] Tabatabaie M. M. , Aliarabi H. , Saki A. A. , Ahmadi A. , and Siyar S. A. , Effect of Different Sources and Levels of Zinc on Egg Quality and Laying Hen Performance, Pakistan Journal of Biological Sciences. (2007) 10, no. 19, 3476–3478, 10.3923/pjbs.2007.3476.3478. 19090175

[25] Yenice E. , Mızrak C. , Gültekin M. , Atik Z. , and Tunca M. , Effects of Dietary Organic or Inorganic Manganese, Zinc, Copper and Chrome Supplementation on the Performance, Egg Quality and Hatching Characteristics of Laying Breeder Hens, Ankara Universitesi Veteriner Fakultesi Dergisi. (2015) 62, 63–68.

[26] Cufadar Y. , Göçmen R. , Kanbur G. , and Yıldırım B. , Effects of Dietary Different Levels of Nano, Organic and Inorganic Zinc Sources on Performance, Eggshell Quality, Bone Mechanical Parameters and Mineral Contents of the Tibia, Liver, Serum and Excreta in Laying Hens, Biological Trace Element Research. (2020) 193, no. 1, 241–251, 10.1007/s12011-019-01698-3. 30941677

[27] Du R. , Qin J. , Wang J. , Pang Q. , Zhang C. , and Jiang J. , Effect of Supplementary Dietary L-Carnitine and Yeast Chromium on Lipid Metabolism of Laying Hens, Asian-Australasian Journal of Animal Sciences. (2005) 18, no. 2, 235–240, 10.5713/ajas.2005.235.

[28] Sirirat N. , Lu J. J. , Hung A. T. , and Lien T. F. , Effect of Different Levels of Nanoparticles Chromium Picolinate Supplementation on Performance, Egg Quality, Mineral Retention, and Tissues Minerals Accumulation in Layer Chickens, The Journal of Agricultural Sciences. (2013) 5, 10.5539/jas.v5n2p150.

[29] Eseceli H. , Degirmencioglu N. , and Bilgic M. , The Effect of Inclusion of Chromium Yeast (Co-Factor II, Alltech Inc.) and Folic Acid to the Rations of Laying Hens on Performance, Egg Quality, Egg Yolk Cholesterol, Folic Acid and Chromium Levels, Journal of Animal and Veterinary Advances. (2010) 9, no. 2, 384–391, 10.3923/javaa.2010.384.391.

[30] Sahin K. , Küçük O. , and Sahin N. , Effects of Dietary Chromium Picolinate Supplementation on Performance and Plasma Concentrations of Insulin and Corticosterone in Laying Hens Under Low Ambient Temperature, Journal of Animal Physiology and Animal Nutrition. (2001) 85, no. 5-6, 142–147, 10.1046/j.1439-0396.2001.00314.x. 11686782

[31] Sahin K. , Ozbey O. , Onderci M. , Cikim G. , and Aysondu M. H. , Chromium Supplementation Can Alleviate Negative Effects of Heat Stress on Egg Production, Egg Quality and Some Serum Metabolites of Laying Japanese Quail, The Journal of Nutrition. (2002) 132, no. 6, 1265–1268, 10.1093/jn/132.6.1265. 12042444

[32] Lien T. F. , Chen K. L. , Wu C. P. , and Lu J. J. , Effects of Supplemental Copper and Chromium on the Serum and Egg Traits of Laying Hens, British Poultry Science. (2004) 45, no. 4, 535–539, 10.1080/00071660400001082. 15484730

[33] Ma W. , Gu Y. , Lu J. , Yuan L. , and Zhao R. , Effects of Chromium Propionate on Egg Production, Egg Quality, Plasma Biochemical Parameters, and Egg Chromium Deposition in Late-Phase Laying Hens, Biological Trace Element Research. (2014) 157, no. 2, 113–119, 10.1007/s12011-013-9875-5. 24338491

[34] Siloto E. V. , Sartori J. R. , Santos T. S. et al., Effects of Chromium Yeast Supplementation on Productive and Metabolic Responses of Laying Hens Fed Diets Containing Different Energy Levels, Revista Brasileira de Zootecnia. (2021) 50, 10.37496/rbz5020200173.

[35] Mashkoor J. , Khan A. , Khan M. Z. , and Hussain I. , Chromium Toxicity and Oxidative Stress in Broiler Chicks and Its Amelioration with Vitamin E and Bentonite, International Journal of Agriculture and Biology. (2016) 18, no. 06, 1103–1108, 10.17957/IJAB/15.0192.

[36] Mathivanan R. and Selvaraj P. , Influence of Dietary Cr on Egg Production and Quality Parameters in Layers, Indian Journal of Poultry Science. (2003) 38, 51–53.

[37] Grashorn M. , Feed Additives for Influencing Chicken Meat and Egg Yolk Color, Handbook on Natural Pigments in Food and Beverages, 2016, London, UK: Woodhead Publishing Limited, 283–302, 10.1016/B978-0-08-100371-8.00014-2.

[38] Molteni C. , La Motta C. , and Valoppi F. , Improving the Bioaccessibility and Bioavailability of Carotenoids by Means of Nanostructured Delivery Systems: A Comprehensive Review, Antioxidants. (2022) 11, no. 10, 10.3390/antiox11101931. PMC9598319 36290651

[39] Olechnowicz J. , Tinkov A. , Skalny A. , and Suliburska J. , Zinc Status is Associated with Inflammation, Oxidative Stress, Lipid, and Glucose Metabolism, The Journal of Physiological Sciences. (2018) 68, no. 1, 19–31, 10.1007/s12576-017-0571-7. 28965330 PMC5754376

[40] Hess S. Y. , Thurnham D. I. , and Hurrell R. F. , Influence of Provitamin A Carotenoids on Iron, Zinc, and Vitamin A Status, HarvestPlus Technical Monograph. (2005) 6.

[41] Abedini M. , Shariatmadari F. , Karimi Torshizi M. A. , and Ahmadi H. , Effects of Zinc Oxide Nanoparticles on the Egg Quality, Immune Response, Zinc Retention, and Blood Parameters of Laying Hens in the Late Phase of Production, Journal of Animal Physiology and Animal Nutrition. (2018) 102, no. 3, 736–745, 10.1111/jpn.12871. 29493020

[42] Klecker D. , Zeman L. , Jelinek P. , and Bunesova A. , Effect of Manganese and Zinc Chelates on the Quality of Eggs, Acta Universitatis Agriculturae et Silviculturae Mendelianae Brunensis. (2002) 50, 59–68.

[43] Torki M. , Zangeneh S. , and Habibian M. , Performance, Egg Quality Traits, and Serum Metabolite Concentrations of Laying Hens Affected by Dietary Supplemental Chromium Picolinate and Vitamin C Under a Heat-Stress Condition, Biological Trace Element Research. (2014) 157, no. 2, 120–129, 10.1007/s12011-013-9872-8. 24347228

[44] Shekhawat K. , Chatterjee S. , and Joshi B. , Chromium Toxicity and Its Health Hazards, International Journal of Advanced Research. (2015) 3, 167–172.

[45] Zhigalenok Y. , Tazhibayeva A. , Kokhmetova S. et al., Hexavalent Chromium at the Crossroads of Science, Environment and Public Health, RSC Advances. (2025) 15, no. 27, 21439–21464, 10.1039/D5RA03104D. 40567474 PMC12188526

[46] Lien T. F. , Chen S. Y. , Shiau S. P. , Froman D. P. , and Hu C. Y. , Chromium Picolinate Reduces Laying Hen Serum and Egg Yolk Cholesterol, The Professional Animal Scientists. (1996) 12, no. 2, 77–80, 10.15232/S1080-7446(15)32493-1.

[47] Sinclair-Black M. , Garcia R. A. , and Ellestad L. E. , Physiological Regulation of Calcium and Phosphorus Utilization in Laying Hens, Frontiers in Physiology. (2023) 14, 10.3389/fphys.2023.1112499. PMC9942826 36824471

[48] Page T. G. , Southern L. L. , Ward T. L. , and Thompson D. L.Jr, Effect of Chromium Picolinate on Growth and Serum and Carcass Traits of Growing-Finishing Pigs, Journal of Animal Science. (1993) 71, no. 3, 656–662, 10.2527/1993.713656x. 8463153

[49] Sahin K. , Sahin N. , Güler T. , and Ertafl O. N. , The Effect of Supplemental Dietary Chromium on Performance, Some Blood Parameters and Tissue Chromium Contents of Rabbits, Turkish Journal of Veterinary and Animal Sciences. (2001) 25, 217–221.

[50] Idowu O. M. , Ajuwon R. O. , Oso A. O. , and Akinloye O. A. , Effects of Zinc Supplementation on Laying Performance, Serum Chemistry and Zn Residue in Tibia Bone, Liver, Excreta and Egg Shell of Laying Hens, International Journal of Poultry Science. (2011) 10, no. 3, 225–230, 10.3923/ijps.2011.225.230.

[51] Adriani L. , Mushawwir A. , Kumalasari C. , Nurlaeni L. , Lesmana R. , and Rosani U. , Improving Blood Protein and Albumin Level Using Dried Probiotic Yogurt in Broiler Chicken, Jordan Journal of Biological Sciences. (2021) 14.

[52] Sedgh-Gooya S. and Torki M. , Influence of Dietary Supplemental Chromium and Magnesium on Performance and Metabolic Parameters of Laying Hens Subjected to Heat Stress, Journal of Applied Animal Research. (2018) 46, no. 1, 1469–1477, 10.1080/09712119.2018.1535436.

[53] Hamidi O. , Chamani M. , Ghahri H. , Sadeghi A. A. , and Malekinejad H. , Effects of Chromium (III) Picolinate and Chromium (III) Picolinate Nanoparticles Supplementation on Growth Performance, Organs Weight and Immune Function in Cyclic Heat Stressed Broiler Chickens, Kafkas Universitesi Veteriner Fakultesi Dergisi. (2017) 22, no. 3, 373–380, 10.29011/2637-9988/100013.

[54] Fawzy M. M. , El-Sadawi H. A. , El-Dien M. H. , and Mohamed W. A. , Hematological and Biochemical Performance of Poultry Following Zinc Oxide and Sodium Selenite Supplementation as Food Additives, Annals of Clinical Pathology. (2016) 4.

[55] Abd El-Hack M. E. , Alagawany M. , Amer S. A. , Arif M. , Wahdan K. M. , and El-Kholy M. S. , Effect of Dietary Supplementation of Organic Zinc on Laying Performance, Egg Quality and Some Biochemical Parameters of Laying Hens, Journal of Animal Physiology and Animal Nutrition. (2018) 102, e542–e549, 10.1111/jpn.12793. 28990706

[56] Huynh U. , Qiao M. , King J. et al., Differential Effects of Transition Metals on Growth and Metal Uptake for Two Distinct Lactobacillus Species, Microbiology Spectrum. (2022) 10, no. 1, e01006–e01021, 10.1128/spectrum.01006-21. PMC8791193 35080431

[57] Yu Q. , Liu H. , Yang K. et al., Effect of the Level and Source of Supplementary Dietary Zinc on Egg Production, Quality, and Zinc Content and on Serum Antioxidant Parameters and Zinc Concentration in Laying Hens, Poultry Science. (2020) 99, no. 11, 6233–6238, 10.1016/j.psj.2020.06.029. PMC7647701 33142541

[58] Habibian M. , Ghazi S. , and Moeini M. M. , Lack of Effect of Dietary Chromium Supplementation on Growth Performance and Serum Insulin, Glucose, and Lipoprotein Levels in Broilers Reared Under Heat Stress Condition, Biological Trace Element Research. (2013) 153, no. 1-3, 205–211, 10.1007/s12011-013-9663-2. 23591960

[59] Arthington J. D. and Ranches J. , Trace Mineral Nutrition of Grazing Beef Cattle, Animals. (2021) 11, no. 10, 10.3390/ani11102767. PMC8532955 34679788

[60] Hossain S. M. , Organic Chromium in Poultry: Metabolic Responses, Effects on Broiler Carcass Composition, Nutrient Composition of Eggs, Poultry Science. (1998) 77, 203–216.

[61] Liu Y. , Liu C. , Cheng J. , Fan W. , Zhang X. , and Liu J. , Growth Performance and Oxidative Damage in Kidney Induced by Oral Administration of Cr (III) in Chicken, Chemosphere. (2015) 139, 365–371, 10.1016/j.chemosphere.2015.07.032. 26207879

